# Huntington's disease: a clinical review

**DOI:** 10.1186/1750-1172-5-40

**Published:** 2010-12-20

**Authors:** Raymund AC Roos

**Affiliations:** 1 Department of Neurology K5Q112, LUMC PO Box 9600, 2300RC Leiden The Netherlands

## Abstract

Huntington disease (HD) is a rare neurodegenerative disorder of the central nervous system characterized by unwanted choreatic movements, behavioral and psychiatric disturbances and dementia. Prevalence in the Caucasian population is estimated at 1/10,000-1/20,000. Mean age at onset of symptoms is 30-50 years. In some cases symptoms start before the age of 20 years with behavior disturbances and learning difficulties at school (Juvenile Huntington's disease; JHD). The classic sign is chorea that gradually spreads to all muscles. All psychomotor processes become severely retarded. Patients experience psychiatric symptoms and cognitive decline. HD is an autosomal dominant inherited disease caused by an elongated CAG repeat (36 repeats or more) on the short arm of chromosome 4p16.3 in the Huntingtine gene. The longer the CAG repeat, the earlier the onset of disease. In cases of JHD the repeat often exceeds 55. Diagnosis is based on clinical symptoms and signs in an individual with a parent with proven HD, and is confirmed by DNA determination. Pre-manifest diagnosis should only be performed by multidisciplinary teams in healthy at-risk adult individuals who want to know whether they carry the mutation or not. Differential diagnoses include other causes of chorea including general internal disorders or iatrogenic disorders. Phenocopies (clinically diagnosed cases of HD without the genetic mutation) are observed. Prenatal diagnosis is possible by chorionic villus sampling or amniocentesis. Preimplantation diagnosis with *in vitro *fertilization is offered in several countries. There is no cure. Management should be multidisciplinary and is based on treating symptoms with a view to improving quality of life. Chorea is treated with dopamine receptor blocking or depleting agents. Medication and non-medical care for depression and aggressive behavior may be required. The progression of the disease leads to a complete dependency in daily life, which results in patients requiring full-time care, and finally death. The most common cause of death is pneumonia, followed by suicide.

## Introduction

The first description by Waters, of a patient with what we now call Huntington's chorea, dates from 1842. But it was not until 1872, after the lecture and description of the disease by George Huntington, that it became known as Huntington's chorea. It is a neurodegenerative disorder passing within families from generation to generation with onset in middle age and characterized by unwanted choreatic movements, behavioral and psychiatric disturbances and dementia [1] For many decades its name remained unchanged, until the nineteen-eighties when, fully aware of the extensive non-motor symptoms and signs, the name was changed to Huntington's disease (HD). In 1983, a linkage on chromosome 4 was established and in 1993 the gene for HD was found [2]. That period marked a tremendous increase in interest in HD and neurogenetic disorders. For the first time, actual premanifest diagnoses could be made and as more diseases involving trinucleotide repeats of CAG were found, HD served as a model for many studies in medicine. CAG (cytosine (C), adenine (A), and guanine (G)), is a trinucleotide, the building stone of DNA. CAG is the codon for the amino acid glutamic. Finding the gene opened new research lines, new models and for the first time a real rationale on the way to treat this devastating disease. Many symptomatic treatments are now available, but there is a need for better, modifying drugs.

## Epidemiology

Huntington's disease is a rare neuropsychiatric disorder with a prevalence of 5-10 per 100,000 in the Caucasian population. In Japan, a much lower prevalence of about one-tenth of prevalence of the Caucasion population is described [3]. Recently, several phenocopies have been described, all of which have an even lower prevalence (see paragraph on differential diagnosis).

## Clinical description

The nuclear symptoms and signs of Huntington's disease (HD) consist of motor, cognitive and psychiatric disturbances. Other less well-known, but prevalent and often debilitating features of HD include unintended weight loss, sleep- and circadian rhythm disturbances and autonomic nervous system dysfunction. The mean age at onset is between 30 and 50 years, with a range of 2 to 85 years. The mean duration of the disease is 17-20 years. The progression of the disease leads to more dependency in daily life and finally death. The most common cause of death is pneumonia, followed by suicide.

### The motor symptoms and signs

The characteristic motor changes are involuntary, unwanted movements. Initially, the movements often occur in the distal extremities such as fingers and toes, but also in small facial muscles. For bystanders these muscle twitches are often invisible or can be explained as nervousness. In daily life, walking becomes unstable and the person can look as if he/she is slightly drunk. Gradually the unwanted movements spread to all other muscles from distal to more proximal and axial. Choreatic movements are present all the time the patient is awake. No single pattern exists, but facial choreatic movements can lead to a continuous movement of facial muscles where for instance an eyebrow is lifted, an eye closed, the head is bent or turned while the tongue is protruded with the lips pouting. The most prominent are the extension movements of the long back muscles. Talking and swallowing gradually become more problematic leading to choking at any time in some patients. In later stages the patient even becomes mute. Dysarthria and dysphagia become very prominent during the course of the disease. All patients develop hypokinesia, akinesia, and rigidity leading to a slower pace of all activities (bradykinesia: slowness of movement) and a severe hesitation in embarking on a movement (akinesia: difficulty in starting movemevents)). The balance between chorea and hypokinesia is determined individually. The extremes are on the one hand the younger patient with an overwhelming rigidity (Westphal variant) and on the other hand the very old patient severely affected in the last stage of the disease with a long duration of illness, bed-bound with rigidity and flexion contractures in the extremities. Dystonia is characterized by slower movements with an increased muscle tone leading to abnormal posture, for instance torticollis, but also rotation of the trunk or limbs. Dystonia (for instance torticollis) can be the first motor sign in Huntington's disease. Other unwanted movements include tics, comparable to the ones seen in Tourette syndrome, but these are fairly rare. Cerebellar signs can appear sporadically, similar to the presence of hypo- and hypermetria. Walking is often described as 'drunk' or 'cerebellar ataxia'-like. Distinguishing between choreatic and ataxic walking is very difficult. Pyramidal signs (Babinski sign) are present incidentally.

The influence of motor disturbance on activities of daily life progresses over time. The presence of hyperkinesia and hypokinesia results in difficulties in walking and standing, and frequently leads to an ataxic gait and frequent falls. Furthermore, daily activities such as getting out of bed, taking a shower, dressing, toileting, cleaning the house, cooking and eating become more and more difficult. Depending on the kind of work the patient does, motor signs will sooner or later interfere with performance, even if psychiatric and cognitive changes are still in the background.

### Behaviour and psychiatric symptoms and signs

Psychiatric symptoms are very frequently present in the early stage of the disease, often prior to the onset of motor symptoms. The percentage of patients with psychiatric signs varies between 33% and 76% depending on the methodology of the study[4]. Because of their impact on daily life, these symptoms and signs usually have a highly negative impact on functioning and on the family [5]. The most frequently occurring sign is depression. The diagnosis is difficult because weight loss, apathy and inactivity also occur in HD. Usually there is low self-esteem, feelings of guilt and anxiety. Apathy is related to disease stage, whereas anxiety and depression are not. Suicide occurs more frequently in early symptomatic individuals and also in premanifest gene carriers. Around the time of the gene test and the stage when independence diminishes are the most risky periods for suicide. Anxiety also occurs frequently (34-61%), sometimes in relation to uncertainty about the start and or the course of the disease. Obsessions and compulsions can disturb the patient's life and also lead to irritability and aggression. Irritability is often the very first sign, in retrospect, but in fact occurs during all stages of the disease [4]. The way irritability is expressed varies enormously from serious disputes to physical aggression. A loss of interest and increasing passive behaviour are seen as part of the apathy syndrome. It can be difficult to discriminate apathy from depression. Pychosis may appear, mainly in the later stages of the disease. In most cases this goes together with cognitive decline. The complete clinical picture is comparable to schizophrenia with paranoid and acoustic hallucinations. In the early stages, hyper-sexuality can cause considerable problems in a relationship. In the later stages hypo-sexuality is the rule.

### Dementia

Cognitive decline is the other main sign of HD and can be present long before the first motor symptoms appear, but can also be very mild in far advanced stages of the disease. The cognitive changes are particularly in relation to executive functions. In normal conditions, cognitive and motor behaviour is goal-directed and planned. Normally individuals are able to distinguish what is relevant and what can be ignored, but patients with HD lose this capability. The patients are no longer able to organise their life or to plan things which in the past were simple. They lose flexibility of mind, and can no longer make mental adjustments. Misjudgements lead to complicated situations, with patients no longer reacting as they did in the past or in a way that the environment expects. Language is relatively spared. Memory certainly becomes impaired, although the semantic memory can be spared to a certain extent. All psychomotor processes become severely retarded [3].

#### Secondary symptoms and signs

From early on, an unintended weight loss has been reported in all patients. As more attention is now paid to this phenomenon, the loss seems to be a little less severe, the cause being diverse. Although it seems logical to think that chorea should play the main role in weight loss, it has been shown that there is no relation between weight loss and chorea or other movement disorders. A relation with the length of the CAG repeat has been described [6]. More practical issues, such as slower functioning, decreased appetite, difficulty handling food and swallowing certainly play a role. But hypothalamic neuronal loss is also a causative factor [7,8].

Attention has only recently been focused on sleep- and circadian rhythm disturbances of patients with HD [9]. Autonomic disturbances can result in attacks of profuse sweating.

### Juvenile Huntington's disease

If the first symptoms and signs start before the age of 20 years, the disease is called Juvenile Huntington's disease (JHD). Behaviour disturbances and learning difficulties at school are often the first signs. Motor behaviour is often hypokinetic and bradykinetic with dystonic components. Chorea is seldom seen in the first decade and only appears in the second decade. Epileptic fits are frequently seen. The CAG repeat length is over 55 in most cases. In 75% of the juveniles the father is the affected parent [10].

### Development of the disease

The course of the life of a person with one parent with Huntington's disease can be divided into an at-risk, a preclinical (A) and a clinical (B) stage. The at-risk stage comes to an end when it is determined whether the person carries the increased CAG repeat on chromosome 4. If he does carry the gene, then he will go through the preclinical and clinical stages until the end.

The clinical course is roughly divided into three parts (Table 1), indicating a decrease in independence and increase in the need for care. The clinical stage with clear manifest signs is preceded by the premanifest gene positive stage, and the transition or phenoconversion phase, when more and more doubt about manifestations of signs emerges. During periods of stress, irrespective of whether this is physical or psychological, it is not uncommon for clinical symptoms or signs to become manifest. These signs can fade away temporarily when the circumstances normalize. In the past, the first symptom was always a motor sign. Dependent on the family's and the doctor's experience with the disease, a diagnosis was suggested. However, over the last 20 years it has become clear [11,12] that psychiatric as well as cognitive changes can be the first signs, many years before motor signs become visible. If the non-motor signs are less specific, it can be very difficult to make a diagnosis. In retrospect, many patients mention a gradual change in behaviour and performance at work. They stayed home for some time with a 'burn-out' or a 'depression'. These signs are rather non-specific and often have a plausible explanation. With improved knowledge about the genetic status and the course of the disease it has become clear that these signs can be the first manifestation of Huntington's disease.

**Table 1:** Stages during the life of a Huntington's disease patient

A. Preclinical stage	
**A1. At-risk stage (50%), one affected parent**	- Anxiousness for the future

	- Uncertainty about carriership

	- Care for affected parent

	

**A2.Gene carrier, premanifest stage**	- Certainty about carriership

	- New position in the family

	- Renewed uncertainty about onset

	- Care for affected parent and own family

	

**A3. Transition phase**	- Strong feelings about changes in cognition

	- Changes in behaviour

	- Changes in motor activity

	- Uncertainty remains

	

**B. Clinical stage**	

**B1. Clinical stage I**	- Presentation first symptoms: neurological, cognitive or psychiatric

	- Chorea most prominent symptom

	- Independent in ADL

	- Burden for the family mainly psychological

	- Rare death, unless suicide

	

**B2 Clinical stage II**	- Motor disturbance more generalised

	- Physical dependence starts

	- Burden for family psychological and physical

	- Death by other cause, suicide, euthanasia

	

**B3. Clinical stage III**	- Severe generalised motor disturbance

	- Almost complete physical dependence

	- Patient completely dependent on care

	- Burden for family mainly physical

	- Death

## Assessments

The clinical assessment of the symptoms and signs of HD is important for patient, family and care-givers. To follow the patient systematically, mainly for research purposes, several scales have been developed. The best known are the Shoulson and Fahn capability scale [13] and the Unified Huntington Disease Rating Scale (UHDRS) [14,15]. The UHDRS consists of a motor, behaviour, cognitive and functional part, preceded by a history and medication scheme. For the behaviour signs a new scale was developed by Craufurd: the Problem Behaviour Scale (PBS)[16]. Other scales, for instance for the quality of life, are also in use. In the European Network for Huntington disease (EHDN: website [17]) a whole set of assessment scales has been devised, which are now in use for over 6,000 patients in Europe.

## Aetiology

Huntington's disease is an autosomal dominantly inherited disease caused by an elongated CAG repeat on the short arm of chromosome 4p16.3 in the Huntingtine gene [2]. This gene codes for the huntingtin protein and, on exon 1, contains the CAG tract. The wild-type contains a CAG repeat, coding for a polyglutamine stretch in the protein at that site in the range 6 to 26. Huntington's disease is associated with 36 repeats or more. Definite clinical manifestation will occur if the number of repeats exceeds 40. The range 36-39 leads to an incomplete penetrance of the disease or to a very late onset. The range between 29 and 35, the so-called intermediate alleles, is unstable, which means that these alleles are prone to changes during reproduction. Copying the gene may lead to mistakes and very often leads to elongation and seldom to shortening. This phenomenon is mainly seen in the male line of reproduction [18].

An inverse correlation has been described between the length of the repeat and the age at onset, determined by the first motor manifestation. The longer the CAG repeat, the earlier the onset. When the disease starts before the age of 20 years, so-called juvenile Huntington's disease (JHD), the repeat often exceeds 55 [5]. The length of the repeat determines about 70% of the variance in age at onset and gives no indication at all about the initial symptom, the course, or the duration of illness. The only correlation now described is the faster weight loss associated with a longer CAG repeat [6]. Anticipation phenomenon is seen in Huntington families in the paternal line of inheritance.

The normal wild-type Huntingtin protein plays a role in synaptic function, is necessary in the post-embryonic period, possibly has an anti-apoptotic function and is possibly protective against the toxic mutant, huntingtin [19]. There is evidence that the mutant form leads to a gain of function as well as to a loss of function. The role of the mutation has been studied in many models: cells, fibroblasts, C. Elegans, drosophila, mice, rat, sheep and monkey. Mice models (more than 10 available) are most commonly used. As neuronal intranuclear and intracytoplasmic inclusions are found, it is still not clear what role they play. Are the inclusions pathogenic in themselves or are they only a side-product of other mechanisms? The inclusions are present in many areas of the brain. The overall pathology, brain atrophy, particularly in the striatum with extensive neuronal loss, is well known [20,21].

## Diagnosis

The diagnosis is based on the clinical symptoms and signs in a person with a parent with proven HD. First, it is obligatory to take a precise history from the person with symptoms followed by a detailed family history. When all information has been obtained the diagnosis is not very difficult, although non-specific clinical pictures can be misleading. Also when the parent is not known or has died due to another cause at a young age, the clinical picture can be difficult to recognise. It is often necessary to request old information in the form of medical records and autopsy reports. The current gold standard is DNA determination, showing a CAG-repeat of at least 36 on the *huntingtin gene *on chromosome 4. Before 1993, a family history with clinical and morphological verification in at least one of the parents or grandparents was obligatory.

The clinical criteria currently necessary are still motor changes with or without psychiatric or cognitive changes. However, in most cases a combination of the three main signs is present. The combination with the family history is sufficient for diagnosis. No imaging, general blood tests or other diagnostic tools are helpful. For all diagnostic tests, it is necessary to obtain informed consent from the patient. This is important because if that person is given a diagnosis of Huntington's disease, then probably many more individuals around the patient will be confronted with an increased risk of Huntington's disease. Extensive studies are underway to detect biomarkers (clinical, blood, MRI) and hence the transition determining parameters [22].

Several studies are now focussing on changes in function and changes in brain imaging (MRI) before clinical overt manifestation is present. It seems that brain volume and brain connections show changes several years before any clinical manifestation is present [23].

## Differential Diagnosis

When chorea is the presenting and most prominent sign, taking a history is the first and most valuable step. The frequently occurring differential diagnoses for motor sign chorea are given in Table 2. In many cases the underlying cause is another general internal disorder or an iatrogenic disorder. Only very few genetically determined disorders are responsible for choreatic syndromes. In about 1% of the cases clinically diagnosed as HD by the clinician, the genetic test does not confirm the diagnosis. These are the so-called phenocopies [24]. Phenocopies are defined by a clinical diagnosis of HD with chorea, psychiatric and or cognitive signs and an autosomal dominant pattern of inheritance or family history. In the last few years, several have been described (Table 3)

**Table 2:** Differential Diagnosis for chorea

Hereditary	- **Huntington's disease**
	- Benign hereditary chorea

	- Neuroacanthocytosis

	- DentatoRubroPallidoLuysianAtrophy (DRPLA)

	- Wilson disease

	

**Rheumatic disorders**	- Sydenham chorea

	- Chorea gravidarum

	

**Drug-induced**	- Neuroleptic drugs

	- Oral anticonceptive drugs

	- Phenytoine

	- Levo-dopa

	- Cocaine

	

**Systemic disorders**	- Systemic Lupus Erythematodes (SLE)

	- Thyrotoxicosis

	- Polycythemia vera

	- Hyperglycemia

	- AIDS

	- Paraneoplastic

**Table 3:** Phenocopy of Huntington's disease (OMIM) [32]

	Mutation	Locus
**1. HDL1**	octapeptiderepeatexpansion *PRNP*-gen	20pter.p12

**2. HDL2**	CTG/CAG-expansion *JPH3*-gen	16q24.3

**3. HDL3**	Not known	4p15.3

**4. SCA17 (HDL4)**	CAG/CAA-expansion *TBP*-gen	6q27

**5. SCA1/2/3**	CAG-expansion *ATXN1/2/3*-gen	6p23, 12q24, 14q24-q31

**6. DRPLA**	CAG-expansion *ATN1*-gen	12p13

**7. Chorea-acanthocytosis**	mutation *VPS13A*-gen	9q

**8. McLeod syndrome**	mutation *XK*-gen	Xp21.2-21.1

**9. NBIA2**	mutation *PLA2G6*-gen	22q13.1

**10. NBIA1/PKAN**	mutation *PANK2*-gen	20p13-12.3

**11. Friedreich ataxia**	GAA-expansion *FXN*-gen	9q13; 9p23-p11

HDL = Huntington Disease-Like; SCA = Spinocerebellar ataxia; DRPLA = DentatoRubroPallidoLuysian Atrophy; NBIA = Neurodegeneration with Brain Iron Accumulation; PKAN = Pantothenate-Kinase-Associated-Neurodegeneration.

## Genetic Counselling

When the gene was localised on chromosome 4 in 1983 [25], premanifest diagnosis became available for the first time using linkage analysis. Linkage analysis provided the applicant with results, initially with a certainty of 93% and later with a certainty of about 98%. When in 1993 the CAG repeat on chromosome 4 was described, real premanifest diagnosis could be given to those at risk of HD. It was the first disease in which this technique became practically available, functioning as an example of how to cope with new questions and problems. A manifest was written by the HD community, a collaboration of scientists, doctors and lay people [26]. The standard procedure was the following: step 1, consultation with a clinical geneticist and preferably in combination with a psychologist and a neurologist. After 4-6 weeks a second consultation (step 2) takes place including blood sampling. After a period of 6-8 weeks a consultation (step 3) with disclosure is planned. Exclusion criteria for the procedure are: age below 18 years, severe psychiatric illness, and external pressure for the applicant. Long discussions took place concerning applicants with a 25% risk at the time of the request. The procedure was extended with the rule that maximal efforts must be taken by the applicant to get a result from the parent with a 50% risk of HD. Finally the 25% at risk applicant can get his test [27].

### Prenatal diagnosis

As the test can be performed on any cell with a nucleus containing DNA, antenatal diagnosis is also possible. Between the 10^th ^and 12^th ^weeks of pregnancy, chorionic villus sampling and between the 15^th ^and 17^th ^weeks amniocentesis can be performed and DNA-testing carried out. The procedure is only initiated if the parents already know their own genetic status to prevent unwanted disclosure for two individuals at the same time. The procedure is embarked on with the intention of ending the pregnancy if the HD gene is found in the embryo. The mother cannot be forced to agree with this conclusion.

If the parents have not yet been genotyped, one can opt for an exclusion test by comparing the genetic status of the embryo with that of the grandparents. In this situation the result is either 0% risk for the foetus, and so the parent keeps his or her 50% status, or 50% risk for the foetus. The foetus has received a chromosome from the affected grandparent, but it is not known to which chromosome the HD gen is coupled. In this case the foetus has a 50% risk, comparable to the parent, and the parents can decide to abort a 50% at risk baby.

During the last decade, preimplantation diagnostics has also been offered in several countries. The procedure starts with in vitro fertilisation. When the embryo is in its eight-cell stage, one cell is removed for DNA testing. The embryo without the elongated CAG repeat is placed in the mother's womb to allow a normal pregnancy to develop. Before starting this procedure, the genetic status of the parent must be known, although not all countries follow this line of thinking [28].

## Management including treatment

Despite the fact that the pathogenesis of HD has still not been resolved and a cure is not available, many therapeutic options are available for treating symptoms and signs with a view to improving quality of life. Although many signs and symptoms can be treated, it is not always necessary to do so. The patient's limitations in daily life determine whether or not drugs are required. Very little evidence is available about the drug or the dosage to prescribe for any signs and symptoms. Drug treatment is, therefore, individualized and based on expert opinion and daily practice.

Treatment consists of drug prescription and non-medication advice. Surgical treatment does not play an important role in HD and will be addressed only briefly.

### Motor signs

Hyperkinesia, or chorea, is treated with dopamine receptor blocking or depleting agents. Most commonly used drugs for chorea (Table 4) are typical or atypical neuroleptics (dopamine receptor blocking) and tetrabenazine (dopamine depleting). The drugs prescribed differ per country. An extensive review of all medication is given by Bonelli [29,30]. The most commonly used drugs for depression and aggression are listed in Table 5. Clozapine and olanzapine are atypical neuroleptics. Both have an antichoreatic effect. Clozapine requires white cell control in the blood and is, therefore, less practical, making olanzapine the preferred drug. The most frequently reported side effects are weight increase and anti-depressive effects. From small case studies some support can be found for prescribing quetiapine, zotepine, ziprasidone, and risperidon. However, only tetrabenazine, a dopamine depleting drug, has been shown in a controlled trial to significantly reduce chorea [31]. The most common side-effects are depression and sedation. There is a long list of drugs without or with only a very limited result, mostly in open case studies: α-tocopherol amantadine, baclofen, cannabidiol, chlordiazepoxide, choline, clonazepam, creatine, deanol, dextromethorphan, fluoxetine, idebenone, ketamine, lamotrigine, levatiracetam, milacemide, minocycline, muscimol, OPC 14117, PUFA, remacemide, riluzole.

**Table 4:** Drug treatment for chorea.

Tiapride	max 600 mg
**Olanzapine**	max 20 mg

Tetrabenazine*	max 200 mg

**Pimozide**	max 6 mg

**Risperidone**	max 16 mg

**Fluphenazine**	max 10 mg

*When tetrabenazine is officially approved per country, this drug will probably become the drug of first choice based on the literature.	

	

**Hypokinesia**	None

(drug dosages vary individually; here maximal dosages are given; these are seldom necessary in practice)

**Table 5:** Drug Treatment for depression (A) and aggression (B)

A. Depression		B. Aggression	
**Citalopram**	max 60 mg	Citalopram	max 60 mg

**Fluoxetine**	max 60 mg	Sertraline	max 200 mg

**Mirtazapine**	max 45 mgr	Olanzapine	max 20 mg

**Valproinezuur**	max 2000 mg	Dipiperon	max 360 mg

**Carbamazepine**	max 1600 mg	Haloperidol	max 10 mg

(drug dosages vary individually; here maximal dosages are given; these are seldom necessary in practice)

Drug treatment for hypokinesia has been tried using antiparkinsonian drugs, but almost always with very disappointing results. In practice, therefore, dopaminergic drug are not prescribed.

To date, despite several claims, no drug is available with any neuroprotective or disease-delaying effect. Disease modifying drugs are developed, but not available. Also embryonic cell implants, still under study, are not proven treatment options at the moment.

Surgical intervention to treat chorea has been described in a few cases. Deep brain stimulation has a place in other movement disorders such as Parkinson disease.

In Alzheimer's disease, anticholinesterase drugs are in use. In Huntington's disease no clinical trials with Rivastigmine or Donepezil are available. In short-term, open studies, no effect was found.

### Psychiatric signs

As depression and aggressive behaviour are the most devastating to family life, the majority of drugs are prescribed for these signs. All advice is based on open studies and expert opinion. (Table 5)

Besides medication, many other care measures are available. It is important to find the right therapy for the right person at the right time. Non-medical interventions available are: physiotherapy, occupational therapy, speech therapy, dietician, psychologist, social worker, and nurse.

During the course of the disease, the patient requires more care, which can also help his/her partner, for example by having a nurse at home to help with showering. The burden for the caregiver can become too heavy and so help must be found in day-care institutions, usually connected to nursing home facilities. In the period that follows the patient moves into a transition phase and eventually a 24-hour care situation. Throughout the whole process of increasing dependency, psychological help is often needed for the caregiver, who has to deal with increasing responsibilities while losing contact with his or her former partner. Partner groups can be very useful. In general, lay organizations play an enormous role in educating caregivers, patients and families.

Medical and non-medical treatment must be individually tailored, as the symptoms and signs differ by person and over time tremendously. Ideally treatment of patients and their families should be organised by a multidisciplinary team. Treatment is intended to improve quality of life. To date, no cure is available unfortunately.

## Future perspectives

Huntington's disease is a physically, psychologically and socially devastating disorder. Knowledge about the disease and care for patients has increased enormously over the last two decades. As the mean duration of illness is more than 17 years, one tends to forget the many years prior to the onset of symptoms during the at-risk and the preclinical periods, or the premanifest period. Huntington's disease is a lifelong disease for both the individual and the family. From the moment the gene was localised in 1983, and particularly after 1993, attention has focussed on the pathophysiological pathway with the aim of developing a therapy. It was the first autosomal dominant disease where premanifest diagnosis became possible and it was the first trinucleotide disease to be described. Consequently, since 1993 many researchers have developed an interest in this disorder. The number of publications has increased enormously.

What is the current perspective? The basic studies mainly focus on the pathophysiology and the search for biomarkers. A better understanding of the pathophysiology will surely lead to drug development to interfere in the pathological process. Drugs that can slow down, delay, or stop the onset of the disease are being sought. The second issue is the search for reliable, early to detect and clinically relevant markers for onset of the end course of the disease. In the search for biomarkers, a very well organised study is underway: TRACK-HD, a multinational study with 366 participants. This will end in 2011 [22]. The Registry study, the main project of the European Huntington Disease Network, also aims to prepare the field for larger studies when drugs become available for human testing.

In parallel with the rational pathway to find solutions to treat this disorder, attention is being paid to finding the best care for all patients and at-risk persons at this point in time. The developments are promising, but one thing is certain: the road to a solution is a long one.

## Competing interests

The author declares that he has no competing interests.
